# Schizophrenia-Like Psychotic Symptoms Associated to Leigh Syndrome

**DOI:** 10.1155/2023/8886555

**Published:** 2023-08-31

**Authors:** F. Jaballah, R. Ben Soussia Nouira, S. Mallouli, H. Boussaid, S. Younes, L. Zarrouk, S. Younes

**Affiliations:** ^1^Neurology Department, Taher Sfar University Hospital-Mahdia, Monastir University, Ksar Hallal, Tunisia; ^2^Psychiatry Department, Taher Sfar University Hospital-Mahdia, Monastir University, Ksar Hallal, Tunisia

## Abstract

**Introduction:**

Leigh syndrome (LS) is a mitochondrial disease characterized by subacute necrotizing encephalomyelopathy with an estimated incidence of 1:40,000 births. The comorbidity of psychotic symptoms noted in mitochondrial and psychiatric diseases has spurred interest in the effects of DNA mutations and psychiatric disorders. *Case presentation*. We report the case of a Tunisian 28-year-old male diagnosed with maternally inherited Leigh syndrome. He presented anxiety and auditory hallucinations, and he reported a vague, unsystematized delusion evolving since 6 months. Significant remission was observed at risperidone 3 mg/day. *Discussion*. The normality of explorations in our case raised the issue of the link between the two diseases, supporting the hypothesis that mitochondrial dysfunction maybe the primary origin of psychotic disorders.

**Conclusion:**

The aim of our work is to study the relations between mitochondrial dysfunction and psychiatric symptoms. Further study of mitochondrial dysfunction in psychiatric disorders is expected to be useful for the development of cellular disease markers and new psychotropics.

## 1. Introduction

Leigh syndrome (LS) is a mitochondrial disease characterized by subacute necrotizing encephalomyelopathy with an estimated incidence of 1 : 40,000 births [1]. Clinically, LS is characterized by psychomotor delay or regression, more or less muscular hypotonia, and brainstem signs [2]. Almost all cases of LS develop in infancy or early childhood and die within several years due to rapidly progressive muscle weakness and respiratory failure [1].

The comorbidity of psychotic symptoms noted in mitochondrial and psychiatric diseases has spurred interest in the effects of DNA mutations and psychiatric disorders. There is growing evidence that mitochondrial dysfunction is linked to the pathophysiology of psychiatric disorders such as schizophrenia [3].

In our study, we report a rare case with LS. The patient has survived to adolescence and presents schizophrenia-like symptoms, such as persecutory delusions and auditory hallucinations. In Tunisia, only one study on this subject has been published by Mnif et al. [2]. Computer-assisted searches on psychotic symptoms associated with LS yielded eight cases. Our work is particularly interesting in view of the limited number of publications and the promising results of our study based on genetic analysis.

## 2. Case Presentation

We report the case of patient who was born for a Tunisian family with a maternally inherited Leigh syndrome (MILS) harboring the mitochondrial T8993G mutation in the ATPase 6 gene. It was the first and the only case in Tunisia that benefited of a genetic research and that was already published [4].

**Figure 1:**
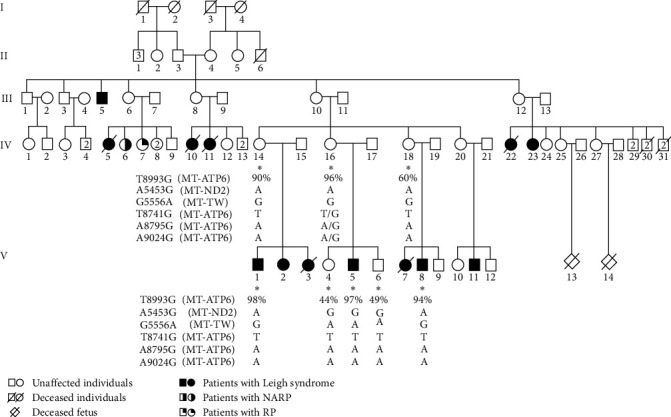
Pedigree of the Tunisian family harboring the T8993G mutation. *Asterisks* indicate the individuals from whom DNA samples were obtained and tested. NARP = neuropathy, ataxia, and retinitis pigmentosa; RP = retinitis pigmentosa.

Actually, The Tunisian family reported here exemplifies a MILS. Three patients in this family and five of their relatives were diagnosed with LS (Figure 1).

After getting informed consent from all the participating family members, total DNA was extracted from peripheral blood using a phenol chloroform protocol. The mitochondrial genes were amplified using a thermal cycler. Quantification of the mutant mtDNA with the mitochondrial T8993G mutation was performed with polymerase chain reaction–restriction fragment length polymorphism analysis [4].

Our patient is a 28-year-old male (V.1, Figure 1) who was born at term to healthy parents after an uneventful pregnancy. He was first admitted at the first month of life for axial and peripheral hypotonia and repetitive seizures. Laboratory investigations revealed raised lactate levels in blood (3.89–8.23 mmol/L; normal, <2 mmol/L) and in CSF (5.7–6.3 mmol/L, normal, <2 mmol/L) with increased blood lactate to pyruvate ratios (36.9–79.9; normal, <25). The EEG and fundus examinations were unremarkable, but EMG revealed myopathic findings. The boy also exhibited psychomotor retardation. At the age of 9 months, he was hospitalized for acute and repeated bronchiolitis, accompanied by seizures. To date, at the age of 22 months, no ataxia or retinitis pigmentosa had been detected. He was treated with antiepileptic drugs for 5 years (sodium valproate: 1,000 mg/day split into two doses).

His history revealed that 6 months ago, gradual onset, the patient developed aggressiveness toward his parents, persecution, mysticism, and difficulty sleeping. Physical examination revealed a patient in a wheelchair. Consciousness was clear, with no confusion. Psychiatric examination revealed low-quality contact with the doctor, with a very anxious and distrustful young man. He presented himself in the exmen room, covering his eyes and ears with a sheet to avoid the sounds he heard. His speech is poor in content, barely comprehensible due to anxiety and auditory hallluciantioins. He reports a vague, unsystematized delusion: he is convinced he is being followed, and “they'll be there to kill him if he doesn't do what they ask for.” He is persecuted by strangers who are collaborating with his parents to kill him. He was overwhelmed by auditory hallucinations and tried constantly throughout the interview to cover his ears. He was anxious and repeated: “they're going to hurt me… they're here… I can hear them screaming my name…” he rubbed his hands and kept moving his legs, telling his mother: “You're their partner in crime, you want to cheat on me … I know all about you.”

The prognosis of psychotic symptoms is not specific to Leigh's disease. it encompasses pathological self-defense reactions. These defensive reactions consist of avoidance or evasive behavior, and sometimes even aggression against the supposed persecutors. In this case, the violent acts are intended to control or eliminate the source of the persecution experienced. They can lead to homicide inside and/or outside the family [2].

The specialized neurological examination showed no clinical worsening. Physical examination was normal. Investigations included routine emergency blood tests (complete blood count, blood sedimentation reaction, liver and kidney function tests, serum electrolytes, and electroencephalogram), which were normal.

**Figure 2:**
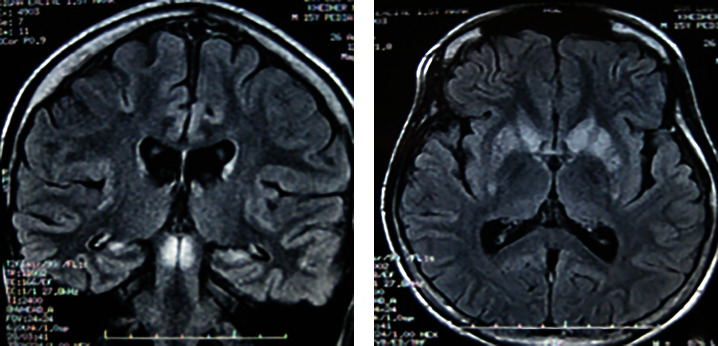
Cranial MRI revealed bilateral and symmetrical hyperintense lesions of the mesencephalon and lenticular and caudate nucleus compatible with LS (transverse view).

Cranial magnetic resonance imaging (MRI) (Figure 2) showed bilateral and symmetrical hyperintense lesions of the mesencephalon and lenticular and caudate nucleus compatible with LS, in line with the normal progression of his disease. Cerebrospinal fluid (CSF) and blood lactate levels were normal.

According to the American Psychiatric Association's Diagnostic and Statistical Manual of Mental Disorders, Fifth Edition (DSM-5), the diagnosis of schizophreniform disorder was adopted. Initially, the patient was under vitamin supplements only (vitamin B1 combined with vitamin E), since no other drugs were available in Tunisia. We prescribed respiridone in small doses (1 mg/day) at first, with follow-up appointments every 2 weeks, combined with a corrector of extrapyramidal effects (biperiden hydrochloride) at a dose of 4 mg/day. Partial remission was observed under these doses, so we opted for increasing the doses in addition 1 mg every 2 weeks until significant remission was observed at risperidone 3 mg.

For an objective assessment of improvement in our case, we used the Psychotic Symptom Rating Scales (PSYRATS): their usefulness and properties in first episode psychosis [5]. The PSYRATS consists of 17 items focusing on hallucinations and delusions. Each item is rated from 0 (absent) to 4 (severe). Initially, our patient's score was 42, with good outcome on treatment: at 2 weeks: 39, at 4 weeks: 35, at 6 weeks: 26 and at 8 weeks: 11 (and the patient began to criticize his delusions and behavioral disorders).

## 3. Discussion

The present study showed phenotypic and genotypic variability. The mutated mtDNA was detected with variable heteroplasmic loads in the blood of all the tested members of this family [6]. Previous case reports have described memory loss, disturbance of consciousness, and visual hallucinations in a few adult patients with LS [7].

Psychotic manifestations of LS are not well documented and highly diverse. This clinical presentation has been described by some authors as a persecutory delusion with auditory and visual hallucinations [2]. Moreover, the types of LS include [8]:

- Early-onset (infantile): The most common form of LS appears before age 2. Providers also call it classical LS or infantile necrotizing encephalopathy. The condition affects boys and girls equally.
- Late-onset (adult-onset): Symptoms appear after age 2 and may not occur until adolescence or early adulthood. Adult-onset LS is rare. The condition affects more males than females. The disease progresses slower than the infantile type.
- Leigh-like syndrome: A person has some symptoms of LS, but imaging scans do not detect signs of the disease.

According to our literature review, no subtype is more vulnerable to the development of psychotic symptoms.

The normality of explorations in our case raised the issue of the relationship between the two diseases: can psychiatric disorders be simple comorbidity or are they secondary to Leigh's syndrome? Regenold et al. [9] have reported that patients diagnosed with mitochondrial disease have high incidences of psychiatric disorders or symptoms such as depression, hallucinations, bipolar disorder, or anxiety. They hypothesized that some psychiatric disorders could be linked to glucose-processing problems involving mitochondria. They studied levels of lactate, a product of extra-mitochondrial glucose metabolism, in two groups of 15 patients, each with schizophrenia or bipolar disorder, as well as in healthy controls. Significantly higher concentrations were observed in the bipolar and schizophrenia patient groups compared with healthy controls.

Moreover, studies have come to the conclusion that mitochondrial dysfunction maybe the primary origin of psychotic disorders [10]. Kung and Roberts [11] studied the pH and lactate levels in the postmortem brains of schizophrenics. They suggest that lactate increases are possibly related to antipsychotic treatment rather than to a primary metabolic abnormality.

A marked decrease in the number and density of mitochondria in the prefrontal cortex and caudate nucleus of postmortem brains of patients with schizophrenia was observed compared with control subjects [12]. Lower numbers of mitochondria were found in patients not taking antipsychotic medication compared with those taking antipsychotic or control medication, suggesting that drug treatment normalizes mitochondrial numbers [13]. In fact, neuroleptics are thought to have suppressive effects on mitochondrial function [14, 15]. The fact that atypical antipsychotics, including quetiapine, have insignificant inhibitory effects on mitochondrial oxidative phosphorylation offers a potential advantage over typical antipsychotics in terms of mitochondrial function. The cost of this drug was very high for the patient's family, and it was available in Tunisian public Hospital.

Evidence for the involvement of mitochondria in the pathophysiology of psychiatric disorders such as schizophrenia is increasing [16]. In fact, the treatment of psychosis in patients with mitochondrial disorders does not differ mainly from the treatment of psychosis in nonmitochondrial patients. There is no consensus on the therapy of psychosis in patients with mitochondrial disorders, even though these are resistant forms of schizophrenia [17].

## 4. Conclusion

The objective of this work is to study the relations between mitochondrial dysfunction and psychiatric disorders. This case illustrates the presentation of psychotic symptoms characterized by the acute onset of delusional and hallucinatory syndrome associated with LS, which suggests a better collaboration between neurologists and psychiatrists for the management of these patients.

The future study of mitochondrial dysfunction in psychiatric disorders should bring clarification as to how nonspecific mitochondrial dysfunction can cause specific symptoms of psychiatric disorders. Further study of mitochondrial dysfunction in psychiatric disorders is expected to be useful for the development of cellular disease markers and new psychotropics.

## Data Availability

All data generated or analyzed during this study are included in this article. Further enquiries can be directed to the corresponding author.
